# Invasive and Ultrasound Based Monitoring of the Intracranial Pressure in an Experimental Model of Epidural Hematoma Progressing towards Brain Tamponade on Rabbits

**DOI:** 10.1155/2014/504248

**Published:** 2014-01-21

**Authors:** Konstantinos Kasapas, Angela Diamantopoulou, Nicolaos Pentilas, Apostolos Papalois, Emmanuel Douzinas, Gregorios Kouraklis, Michel Slama, Abdullah Sulieman Terkawi, Michael Blaivas, Ashot Ernest Sargsyan, Dimitrios Karakitsos

**Affiliations:** ^1^Department of Neurosurgery, Athens General Hospital “G. Gennimatas”, 11527 Athens, Greece; ^2^2nd Department of Surgery, Athens Medical School, Laiko University Hospital, 11527 Athens, Greece; ^3^Intensive Care Unit, Athens General Hospital “G. Gennimatas”, Athens, Greece; ^4^Experimental Research Center ELPEN, 19009 Athens, Greece; ^5^3rd ICU Department, Athens Medical School, Evgenideio University Hospital, 15128 Athens, Greece; ^6^ICU, Amiens University Hospital, 80054 Amiens, France; ^7^Department of Anesthesiology, University of Virginia Health System, Charlottesville, VA 22903, USA; ^8^Department of Anesthesiology, King Fahad Medical City, Riyadh 11525, Saudi Arabia; ^9^Department of Internal Medicine, School of Medicine, University of South Carolina, Columbia, SC 29225, USA; ^10^Wyle Science, Technology & Engineering Group/NASA Bioastronautics, Houston, TX 77058, USA

## Abstract

*Introduction*. An experimental epidural hematoma model was used to study the relation of ultrasound indices, namely, transcranial color-coded-Doppler (TCCD) derived pulsatility index (PI), optic nerve sheath diameter (ONSD), and pupil constriction velocity (*V*) which was derived from a consensual sonographic pupillary light reflex (PLR) test with invasive intracranial pressure (ICP) measurements. *Material and Methods*. Twenty rabbits participated in the study. An intraparenchymal ICP catheter and a 5F Swan-Ganz catheter (SG) for the hematoma reproduction were used. We successively introduced 0.1 mL increments of autologous blood into the SG until the Cushing reaction occurred. Synchronous ICP and ultrasound measurements were performed accordingly. *Results*. A constant increase of PI and ONSD and a decrease of *V* values were observed with increased ICP values. The relationship between the ultrasound variables and ICP was exponential; thus curved prediction equations of ICP were used. PI, ONSD, and *V* were significantly correlated with ICP (*r* ^2^ = 0.84 ± 0.076, *r* ^2^ = 0.62 ± 0.119, and *r* ^2^ = 0.78 ± 0.09, resp. (all *P* < 0.001)). *Conclusion*. Although statistically significant prediction models of ICP were derived from ultrasound indices, the exponential relationship between the parameters underpins that results should be interpreted with caution and in the current experimental context.

## 1. Introduction

The invasive measurement of intracranial pressure (ICP) is an established neuromonitoring tool in patients with traumatic brain injury (TBI) [1–6]. Although the insertion of an intracranial catheter remains the standard method for diagnosing intracranial hypertension, noninvasive monitoring techniques such as computed tomography (CT) scan of the head, ophthalmoscopy, and transcranial color-coded Doppler (TCCD) have been applied on head injured patients in the intensive care unit (ICU) [4, 5, 7]. Apart from TCCD, the development of multipurpose ultrasound systems has facilitated the assessment of optic nerve sheath diameter (ONSD) as a surrogate measure of ICP and recently the recording of pupillary light reflex (PLR) when visual access to the pupil is impeded [4–10].

Although not yet established, the aforementioned ultrasound methods have been gradually integrated in neuromonitoring strategies due to the availability of ultrasound systems which allowed fast and by-the-bed evaluation in the ICU. Ultrasound methods have various limitations as they remain largely operator-dependent, while the presence of inadequate acoustic windows, especially in the case of TCCD, may prevent their application in all cases [4–10]. Our group has previously suggested the significance of combined measurements of the Doppler derived pulsatility index (PI) in the middle cerebral artery (MCA) and ONSD recordings as surrogate measures of ICP in patients with TBI [4, 5]. PI is calculated by the equation: PI = systolic flow velocity (FVs)—diastolic flow velocity (FVd)/mean flow velocity (FVm) and is a well-known measure of vascular impedance [5, 7]. In previous experimental models of TBI progressing towards brain tamponade and thus cerebral circulatory arrest (CCA), increased PI thresholds over 2 were associated with loss of autoregulation and critically reduced cerebral blood flow [11–16]. CCA can be identified by TCCD as the former is characterized by the presence of systolic spikes or oscillating flow [15, 16]. However, the progression of a brain injury to loss of cerebral autoregulation and finally to CCA may indeed follow variable pathophysiologic pathways dependent on the underlying disorder (i.e., stroke, diffuse brain edema, hematoma, etc.). Moreover, the relationship of noninvasive neuromonitoring indices such as the PI, ONSD, and sonographic PLR to invasive ICP measurements remains a debatable issue in patients with TBI [1–11].

In this experimental study, we have used a previously established model of TBI in rabbits which has been developed by our group [17]. In the aforementioned model, a 5-Fr balloon-tipped catheter is placed in the epidural space of rabbits, while gradual infusion of autologous blood results in the reproduction of a mass effect due to the implanted epidural hematoma [17]. In this model, we have invasively evaluated the ICP, during the epidural hematoma formation until the Cushing reaction was established. Simultaneous combined recordings of ultrasound neuromonitoring indices, namely, PI, ONSD, and pupil constriction velocity (*V*) during a consensual PLR test were performed. The end-point was to study the possible relation of the ultrasound neuromonitoring indices to the invasive ICP measurements.

## 2. Materials and Methods

All animal experiments were approved by the veterinary authorities of East Attica region and were in accordance with European Union regulations and the principles of the Helsinki Declaration. The animals had free access to daily food and drink. Twenty-two adult New Zealand white rabbits (10 males, 3 ± 0.3 Kg) participated in the study. Two animals died during intubation, and thus 20 animals were included in the final analysis.

In all rabbits, we facilitated the reproduction of an epidural hematoma that consequently resulted in brain tamponade [17]. Briefly, the central auricular artery and marginal ear vein were cannulated. Anesthesia was induced with intravenous Pentobarbital (Dolethal 200 mg/mL) (2 mL diluted in 8 mL N/S) 1–1.5 mL and maintained with 0.3–1 mL/20 min, while for analgesia Fentanyl 0.5 mg/10 mL was infused and maintained at 0.02 mg/kgr/20 min. Adjustments of the anesthesia depth were guided according to the pedal withdrawal, the ear pinch and the eye lid reflexes. Tracheal intubation was performed in 18 animals using a 2.5–3 mm diameter tube. Tracheotomy was performed in two animals which could not be intubated. All animals were ventilated with a volume-controlled ventilator (Tiberius 19, Drogerwerk AG, Lobeck, Germany) using low tidal volumes (set at 5–10 mL/kg) and adjusted respiratory rates to maintain the carbon dioxide pressure in arterial blood (PCO2) between 32 and 35 mmHg.

**Figure 1:**
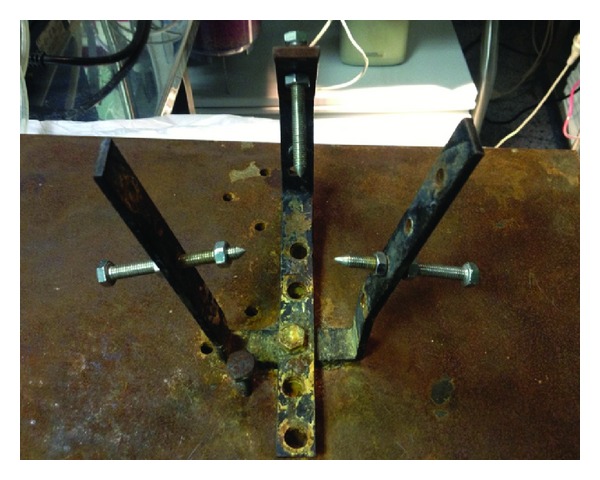
The offhand three-dimensional frame used to stabilize the head of the rabbit.

**Figure 2:**
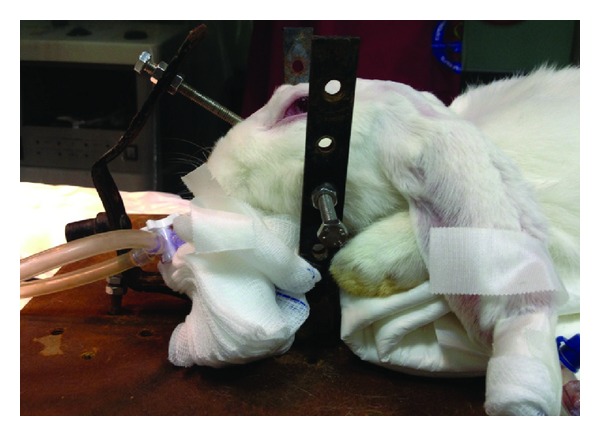
An intubated rabbit stabilized by the three-dimensional frame.

**Figure 3:**
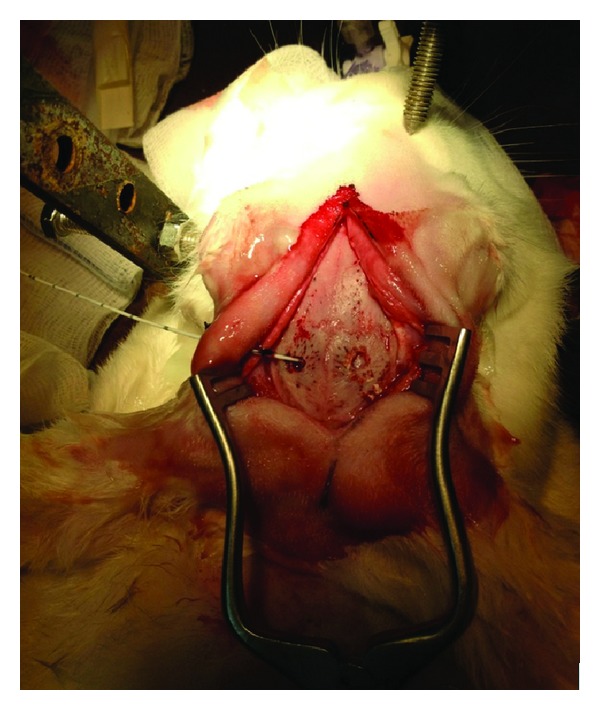
Intraprocedural image of the rabbit's skull depicting the two burr holes and the insertion of the fiberoptic Camino catheter.

The head of the animal was stabilized by means of an offhand three-dimensional frame almost similar to the 3-point Mayfield system used in neurological procedures (Figures 1 and 2). This was performed to facilitate the surgical process and the consequent ultrasound measurements. Ten minutes after the administration of anesthesia, a middle-line incision was performed and a burr hole approximately 1 cm lateral to the sagittal suture and 1 cm posterior to the coronal suture of the animal's skull was also performed bilaterally. In the first burr hole, the dura matter was incised carefully with a thick needle and a fiberoptic catheter (Camino Laboratories, San Diego, CA, USA) that was connected to a dedicated monitor was placed in the parenchyma, after being calibrated in the air for continuous ICP monitoring (Figure 3). In the contralateral burr hole, a 5F Swan-Ganz balloon-tipped catheter was placed in the epidural space for the reproduction of the epidural hematoma. The volume of the cranial vault was measured by calculating the volume of an injected hydrophilic gel that was required to fill the intracranial vault [17].

### 2.1. Study Protocol

When the experimental setup was completed, reference values of ICP, ONSD, TCD, and *V* were recorded taking into consideration that the volumes of the fiberoptic catheter and the Swan-Ganz balloon catheter were 0.1 and 0.3 mL, respectively. The aforementioned values were entered as baseline values in the statistical analysis. Next, we gradually and successively introduced 0.1 mL increments of autologous blood into the Swan Ganz balloon-tipped catheter that was placed in the epidural space (0.1 mL, 0.2 mL, 0.3 mL, etc.) until the Cushing reaction occurred. Four rabbits exhibited a Cushing reaction at 0.6 to 0.8 mL of infused autologous blood, while the remaining 16 exhibited this reaction when a total volume of 1 mL was reached. Thus, we used the latter as the higher volume reference value of autologous blood infusion in the statistical analysis. We maintained the Cushing reaction for 2-3 minutes in all animals to facilitate a diagnosis of CCA by means of TCCD (see below). Next, the balloon was deflated accordingly.

At each stage of the formation of the epidural hematoma, several minutes (5–10 min) were given to achieve a “steady” state as most animals required additional anesthesia to maintain deep sedation, while intravenous infusion of small amounts of normal saline (10–80 mL) was also necessary to preserve an adequate systolic pressure. During this process, continuous invasive recordings of the ICP were performed, while heart rate and systemic blood pressure were monitored by means of an invasive arterial line which was inserted in the central auricular artery and connected to a dedicated hemodynamic monitor (Edwards monitor, Edwards Lifesciences, Irvine, CA). Electrocardiograms were recorded accordingly. Body temperature was recorded with a rectal temperature probe. At the end of the above-mentioned “steady” state (around 5–10 minutes following each infusion), simultaneous ICP and ultrasound measurements were recorded and included in the analysis. Two sets of three bilateral ultrasound measurements of each ultrasound parameter (PI, ONSD, and *V*) were performed at each stage of the reproduction of the epidural hematoma and average values were used in the final analysis. Five animals immediately expired after the completion of the experiment, while the remaining 15 were killed with a bolus dose of thiopental. In all animals, the skull was opened and both the dura and the brain were examined for possible procedural lesions that could confound the interpretation of results.

### 2.2. Ultrasound Measurements

All measurements were performed by one experienced operator to minimize bias. An M-Turbo ultrasonographic system (SonoSite, Bothell, WA) equipped with a high-frequency linear transducer was used for the ONSD and PLR measurements, while a 2.5 MHz wide-phase array transducer with an appropriate software program was utilized to perform TCCD measurements.

**Figure 4:**
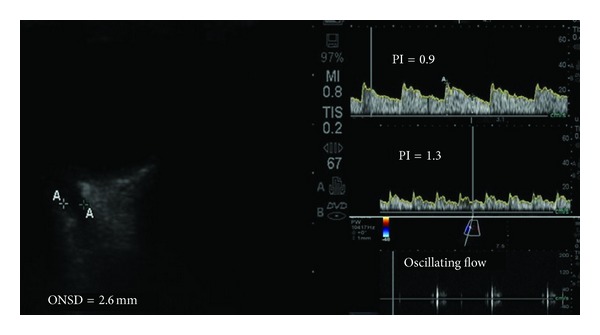
(Left panel) Ultrasound measurements (volume of autologous blood volume infusion = 0.8 mL) by means of the “dark stripe” method depicting an increased ONSD of 2.6 mm in a rabbit with increased ICP (30 mm Hg). (Right panel) TCCD waveforms depicting sequentially: a normal PI (top, volume of autologous blood infusion = 0.1 mL, ICP = 7 mm Hg), an increased PI of 1.3 (middle, volume of autologous blood infusion = 0.6 mL, ICP = 21 mm Hg, and oscillating flow soon after the occurrence of the Cushing reaction (bottom, volume of autologous blood infusion = 1 mL, ICP =40 mm Hg).

TCCD recordings were performed by accessing the anterior and posterior temporal TCCD windows bilaterally [4, 5, 15, 16]. PI values (PI = FVs−FVd/FVm) derived from the Doppler waveform at the area of the insonated middle cerebral artery (MCA) were registered accordingly (Figure 4). The recordings were technically difficult due to the small size of the animal's head and the on-going surgical process. However, the animal's skull was thin, while adjustments of the probe's frequency and intensity (i.e., adjusting the mechanical index) facilitated TCCD recordings in all cases. CCA was diagnosed when systolic spikes or oscillating flow were evident in the MCA as previously described in the literature [4, 5, 15, 16]. CCA was registered on Doppler recordings when the gradually expanding epidural hematoma has created brain tamponade due to its mass effect; moreover, CCA coincided with the occurrence of the Cushing reaction in all animals.

ONSD was measured by using an oblique axial view to access the optic nerve head 2 to 3 mm posterior to the papilla as previously described in the literature [4, 5, 8]. The probe was placed on the upper eyelid and the plane bypassed the anterior structures focusing on the optic nerve head. Unfortunately, the sonographic differentiation (contrast) between the nerve proper and the arachnoid (cerebrospinal fluid space) was not possible. This was mainly due to the fact that in rabbits the optic nerve's size is small and its course rather is oblique. Hence, the ONSD was measured as a “dark stripe” behind the globe (Figure 4). Past studies have suggested increased ONSDs which correlated with invasive ICP measurements and brain computed tomography (CT) findings in patients with severe TBI [4, 5].

**Figure 5:**
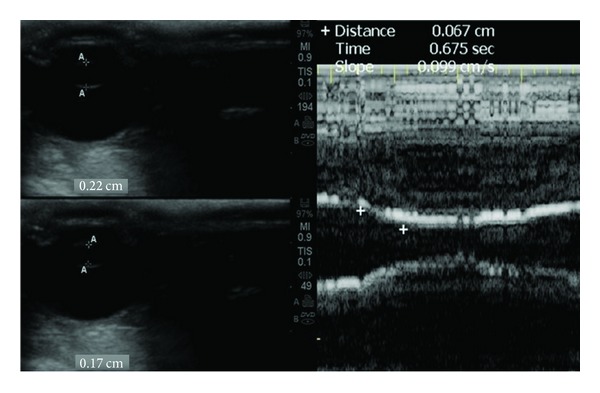
(Left panel) Normal pupillary size at relaxation (top) and following a consensual PLR (bottom) on B-mode. (Right panel) Normal pupil constriction velocity which is calculated by the slope of the M-mode (*V* = 0.09 cm/s in this example) as the latter can be easily placed on the pupil's center when performing a consensual PLR (volume of autologous blood infusion = 0.1 mL, ICP = 8 mm Hg).

The sonographic examination of pupil's diameter and reactivity to light is a new alternative method to standard pupillometry. Past studies have suggested a relationship, particularly in TBI patients with a mass effect, of ICP and *V* when performing a consensual PLR test [18]. The sonographic method was initially developed for the U.S. Space Program and is not currently standardized for clinical use [19]. In this study, a consensual PLR was elicited with contralateral transillumination through the eyelids of the rabbits with both eyes closed. The PLR ultrasound test was conducted with a linear array probe at the highest available frequency using a coronal view through the upper eyelid. In rabbits, the pupil's size is relatively large compared to the size of other ocular structures, and thus it was detectable on B-mode. Finally, M-mode recordings were focused on the two-dimensional images of the pupil and were used to evaluate the pupil's *V* by measuring the slope of the elicited consensual PLR as demonstrated in Figure 5.

### 2.3. Statistical Analysis

Data are presented as mean ± standard deviation (SD), while 95% confidence intervals (CI) were calculated accordingly. Differences between the values of the studied neuromonitoring indices (PI, ONSD, and *V*) at baseline compared to our higher volume reference value (1 mL) were evaluated by paired *t*-test. The relationship between each ultrasound parameter to the invasive ICP measurements was assessed for linearity using pertinent scatter plots, while the strength and direction of the relationship were evaluated by means of Pearson's correlation coefficient [20, 21]. To evaluate the relative impact of a predictor ultrasound variable (PI, ONSD, and *V*) on a particular outcome (ICP), we used linear regression analysis and therefore model-driven prediction equations were formulated accordingly [20, 21]. Due to the fact that the aforementioned relations of predictor ultrasound variables and ICP were exponential rather than straight linear, we used the exponential curve equation: (log⁡(*Y*) = *b*0 + *b*1∗*X*), where “*Y*” is the estimated value (i.e., ICP), “*b*0” is the *y* intercept, “*b*1” is the slope of the line, and “*X*” is the measured value (PI, ONSD, and *V*). The resulted model-driven prediction equations were evaluated accordingly by goodness of fit [20, 21]. All tests were two-sided and a *P* value ≤ 0.05 was considered as statistically significant. Statistical analysis was performed using MedCalc 12 software.

## 3. Results

No procedure-related lesions were found on the dura or the brain of the rabbits after opening the skulls at the end of the experiment. The estimated intracranial vault volume was approximately 20 mL. Four rabbits exhibited a Cushing reaction at 0.6 to 0.8 mL of infused autologous blood, while the remaining 16 exhibited this reaction when a total blood infusion volume of 1 mL has been reached. The studied ultrasound neuromonitoring indices and other parameters at baseline versus at volume 1 mL of epidural hematoma are presented on Table 1. ICP, ONSD, and PI were all significantly increased at 1 mL volume of epidural hematoma compared to baseline values. PI values at the higher volume (1 mL) of the experimental mass effect (brain tamponade, ICP > 40 mm Hg) far exceeded 7 and oscillating flow was the main finding which confirmed CCA, while pupils were fixed, dilated, and unresponsive to light (*V* = 0 cm/s).

**Figure 6:**
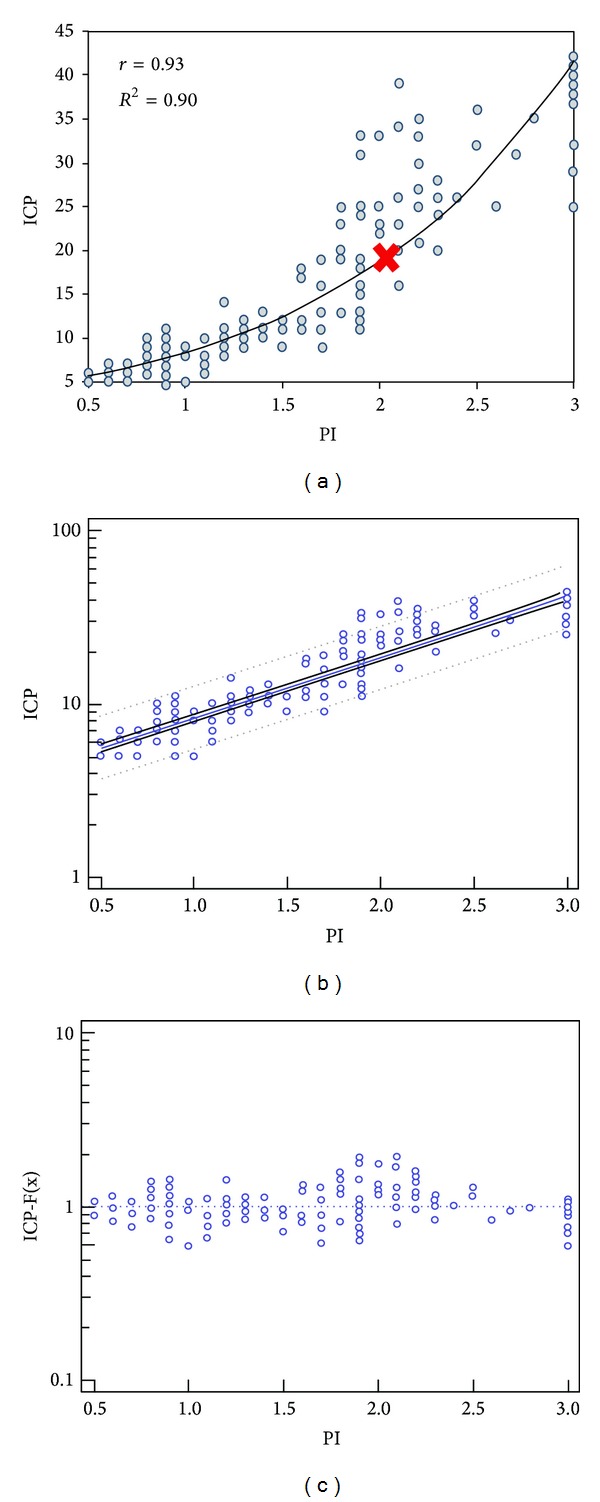
(a) Pulsatility index (PI) plotted versus the ICP (mm Hg); the scatter plot displays a positive exponential relation between the two parameters rather than an exact linear relation. (b) Regression analysis model: the “dots” represent the actual measured values, while the blue line in the middle represents the estimated values by the model and the dashed lines represent 95% confidence intervals. (c) Goodness of fit for the model-driven prediction equation calculating the proximity of the predicted values (dots) to the actual measured values (dashed blue line).

**Figure 7:**
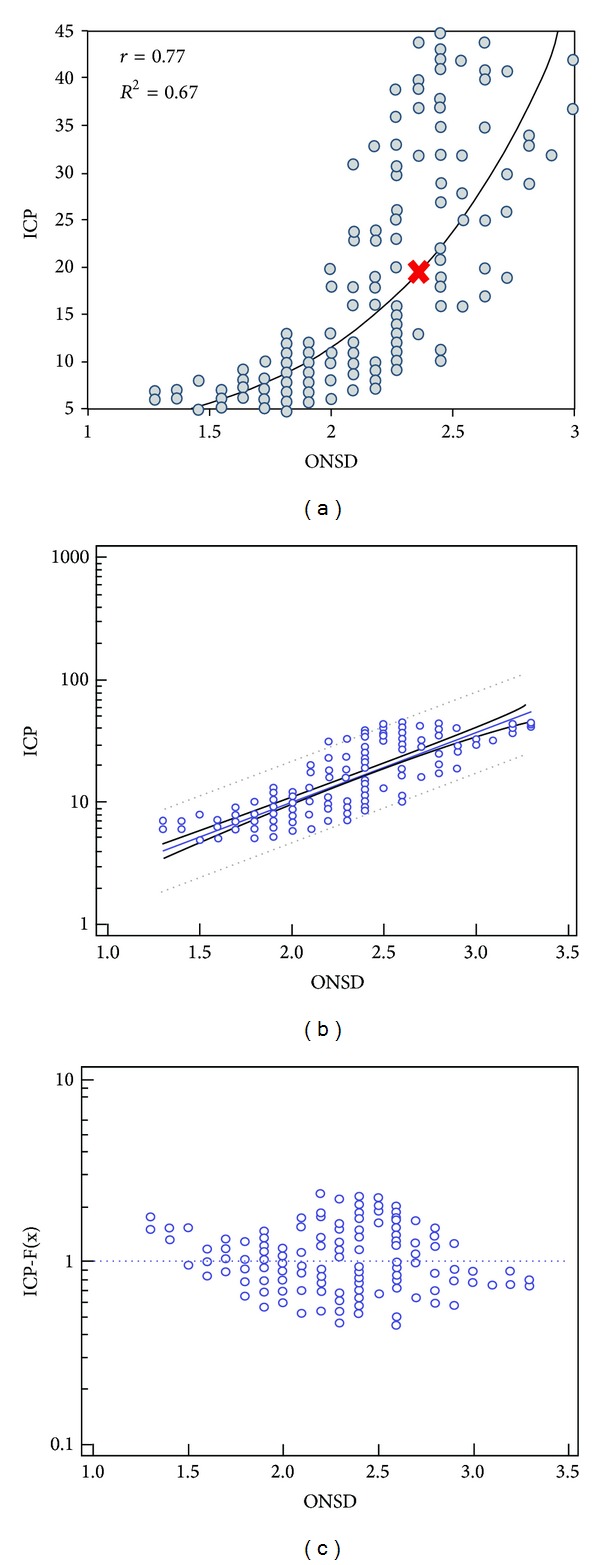
(a) Optic nerve sheath diameter (ONSD) in mm plotted versus the ICP (mm Hg); the scatter plot displays a positive exponential relation (slightly different compared to the PI plot) between the two parameters rather than an exact linear relation. (b) Regression analysis model: the “dots” represent the actual measured values, while the blue line in the middle represents the estimated values by the model and the dashed lines represent 95% confidence intervals. (c) Goodness of fit for the model-driven prediction equation calculating the proximity of the predicted values (dots) to the actual measured values (dashed blue line).

**Figure 8:**
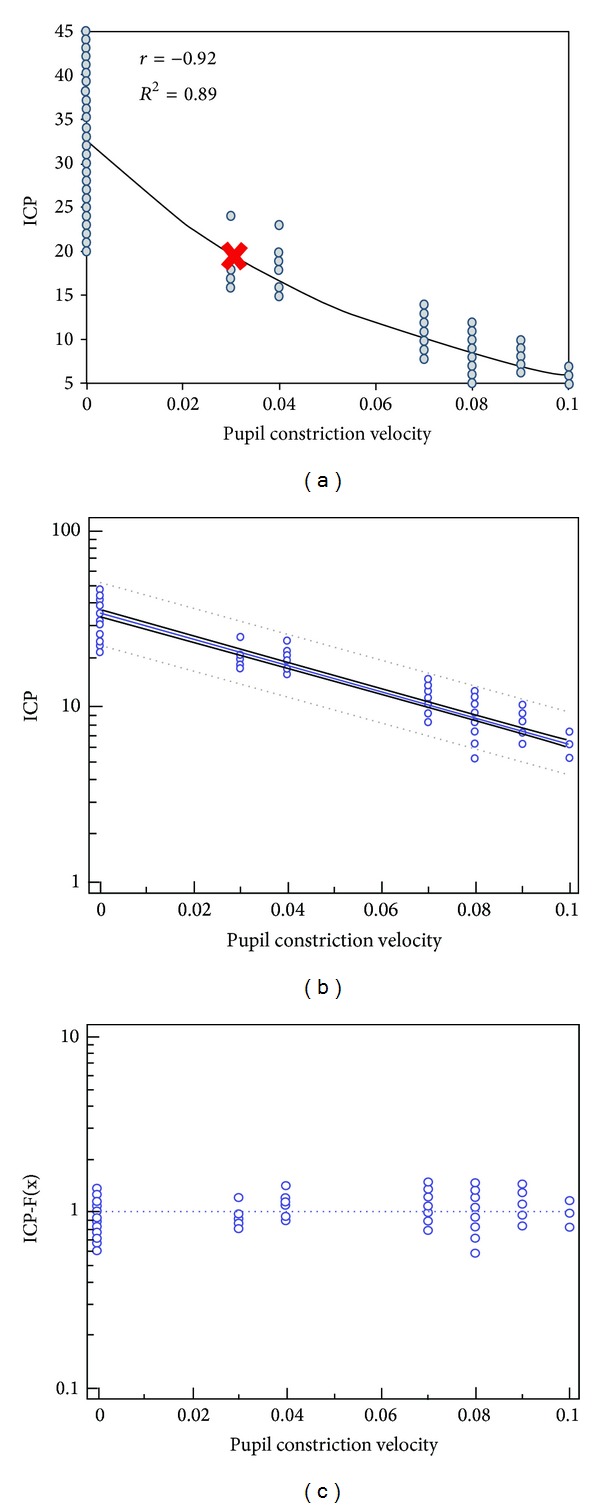
(a) Pupil constriction velocity (*V*) in cm/s plotted versus the ICP (mm Hg); the scatter plot displays a negative exponential relation (red ribbon corresponds to ICP = 20 mm Hg) between the two parameters rather than an exact linear relation. (b) Regression analysis model: the “dots” represent the actual measured values, while the blue line in the middle represents the estimated values by the model and the dashed lines represent 95% confidence intervals. (c) Goodness of fit for the model-driven prediction equation calculating the proximity of the predicted values (dots) to the actual measured values (dashed blue line).

The relationship of the ultrasound neuromonitoring indices with the invasive ICP measurements was studied by regression analysis models as previously mentioned. PI values were plotted versus the ICP and exhibited an exponential relation (Figure 6(a)). As the volume of the epidural hematoma and consequently ICP values gradually increased, a constant increase in PI values was evident until the point of brain tamponade when oscillating flow was observed (no PI values were registered as these far exceeded the ultrasound scale used in the analysis). Notably, the distribution of PI data appeared to be more scattered for ICP values over 20 mm Hg compared to values less than 20 mm Hg. An overall strong positive relationship between PI and ICP was observed (*r* = 0.93; 95% CI 0.91 to 0.95; *P* < 0.001; Figure 6(b)). The model-driven prediction equation of ICP for PI was log (ICP) = 0.63 + 0.30 × PI. The prediction equation was further confirmed by goodness of fit (coefficient of determination *r* ^2^ = 0.84 ± 0.076; *P* < 0.001; Figure 6(c)). Accordingly, ONSD values were plotted versus the ICP and showed a gradual increase as the volume of the epidural hematoma increased. ONSD values also showed an exponential relation to the ICP values (slightly different compared to the PI plot), while an overall strong positive relationship was evident (*r* = 0.75; 95% CI 0.68 to 0.80; *P* < 0.001; Figures 7(a) and 7(b)). The distribution of ONSD data exhibited a rather different pattern compared to PI data as the former also increased gradually but with a “step-like” manner (Figures 6(a) and 7(a)). The model-driven prediction equation of ICP for ONSD was log (ICP) = 0.27 + 0.35 × ONSD. The latter was further evaluated by goodness of fit (coefficient of determination *r* ^2^ = 0.62 ± 0.119; *P* < 0.001; Figure 7(c)). Finally, the pupil constriction velocity (*V*) showed an exponential relation to ICP as well, while the pattern of distribution of *V* data resembled the ONSD scatter plot (Figure 8(a)). Higher ICP values resulted in slower pupil contraction during the consensual PLR test, and thus *V* was gradually decreased. A strong negative relationship between *V* and ICP values was evident (*r* = −0.87; 95% CI −0.90 to −0.83; *P* < 0.001; Figure 8(b)). The model-driven prediction equation of ICP for *V* was log⁡(ICP) = 1.27 + (−0.45) × *V* that was further confirmed by goodness of fit (coefficient of determination *r* ^2^ = 0.78 ± 0.09; *P* < 0.001; Figure 8(c)). In conclusion, statistically significant model-driven prediction equations of ICP derived from all three studied ultrasound neuromonitoring indices were registered. Using all three ultrasound indices to calculate one combined model-driven prediction equation of ICP was not possible due to the small size of the study group and the limited total number of ultrasound measurements.

## 4. Discussion

Invasive ICP evaluation is a gold standard neuromonitoring method in patients with severe TBI. ICP should be monitored in all patients with postresuscitation Glasgow Coma Scale <8 and an abnormal brain CT scan. ICP monitoring is also indicated in patients with severe TBI with a normal brain CT scan when two or more of the following features are present upon admission: age over 40 years, unilateral or bilateral motor posturing and systolic blood pressure <90 mmHg. For other patients the indications for invasive ICP monitoring remain debatable [1–5]. Interestingly, a recent multicenter study in patients with severe TBI has suggested that care focused on maintaining monitored ICP at 20 mm Hg or less has not shown as superior to care based on imaging and clinical examination [22]. Complications related to invasive ICP measurements such as hemorrhage and infection can be avoided by the application of noninvasive neuromonitoring methods. In the ICU, invasive ICP monitoring is contraindicated in patients receiving anticoagulants or having thrombocytopenia as well as in scalp or cerebral infection, while lack of neurosurgical expertise is another usual problem [1–3]. Thus, neuromonitoring strategies may indeed be changing in the near future as intensivists and neurosurgeons are gradually driven away from ICP based diagnostic and therapeutic practices to cerebral perfusion pressure (CPP) related clinical strategies. Apart from ultrasound techniques, several other noninvasive methods which estimate ICP based on physiological or anatomical brain features have been described including CT and magnetic resonance imaging, near-infrared spectroscopy, and visual-evoked potentials [1–3]. Ultrasound neuromonitoring techniques are becoming increasingly popular in the ICU setting due to the availability of multipurpose ultrasound systems and the rapid and by-the-bed nature of the sonographic evaluation. However, the application of ultrasound neuromonitoring techniques on patients with TBI is not yet established or standardized [1–5].

In this experimental study, we have reproduced an epidural hematoma model to study the relation of invasive ICP measurements with simultaneous recordings of ultrasound neuromonitoring indices. The gradually expanding hematoma created a mass effect which needed to reach at a volume of 1 mL to produce a Cushing reaction in the vast majority of the studied animals. In our previously published study, the Cushing reaction was achieved at lower hematoma volumes (0.5-0.6 mL) [17]. This discrepancy may be explained by the fact that in this study the experimental setting was modified as all animals were intubated and mechanically ventilated (deeper sedation); moreover the volume of the cranial vault measured in this study was larger compared to the one measured in the previous one (20 versus 13 mL). The current results indicated statistically significant model-driven prediction equations of ICP derived from the ultrasound neuromonitoring indices, namely, PI, ONSD, and *V*. However, the exponential relationship of all ultrasound data to the ICP measurements (Figures 6, 7, and 8) underpins that the current results should be interpreted with caution and in the context of the specific experimental scenario.

TCCD is a specialized examination which has advantages over the conventional transcranial Doppler examination by showing the images of the intracranial anatomy and arteries by duplex B-mode, while still having the capacity to measure velocities with Doppler. PI is derived from the TCCD waveform as mentioned in previous paragraphs. TCCD may detect alterations of cerebral blood flow caused by elevated ICP and hence identify patients who are at risk of developing cerebral ischemia and/or edema in the early phases of TBI [1–5]. However, absent sonographic windows may be present in 5 to 13% of cases [16]. Absolute pulsatility is difficult to assess by ultrasound as the amplitude of the pulsatile blood flow velocity is dependent on the angle of insonation. Also, it is well known that TCCD flow velocities do not correspond to cerebral blood flow per se [11–16]. Previous reports have linked the PI with distal cerebrovascular resistance suggesting an increasing PI as a reflection of an increasing resistance and vice versa [23, 24]. Other studies have reported that the aforementioned link was weak suggesting thus that the PI is not an absolute measure of cerebrovascular resistance [25, 26]. Previously published animal models in which cerebrovascular resistance was manipulated in a controlled manner under different physiologic conditions such as an increase in arterial PCO2 or by a decrease in CPP suggested that the PI cannot be interpreted merely as an index of cerebrovascular resistance but in the context of combined changes of cerebrovascular resistance and compliance of large cerebral arteries [11–14, 26]. Our results indicated a gradual and constant increase of PI with higher ICP which is in accordance with previous reports [11–14]. Notably, the distribution of PI data was more scattered in ICP values over 20 mm Hg. This might be explained by a loss of cerebral autoregulation above the aforementioned ICP values as other groups have previously reported [11–14, 26]. The high correlation of PI values to the invasive ICP measurements in this study might be attributed to the specific experimental pathophysiologic scenario: the reproduction of the epidural hematoma has created a mass effect which has gradually progressed to brain tamponade. Taking into account the small volume of the rabbit's brain and cranial vault, the expanding epidural hematoma resulted in a direct mass effect on the circle of Willis. Thus, blood flow in the insonated MCA was affected in a straightforward manner. However, the pathophysiology of TBI may be extremely variable (i.e., hematoma, edema, ischemia, loss of autoregulation, etc.) in real time clinical scenarios [4, 5, 11–14, 26]. Despite the aforementioned inherent and technical TCCD limitations, PI remains a useful neuromonitoring variable when individual patient and physiologic features are taken into account in order to make context-relevant use of its measurements in various clinical scenarios [1–5].

Our results indicated that ONSD measurements were positively correlated with the increments of ICP. ONSD measurement is performed as a standalone ultrasound examination of eye and orbit in association with known, suspected, or anticipated ICP elevation. The protocol is reasonably simple to allow fast bedside evaluation in acute conditions with rapidly rising ICP (i.e., TBI) [4, 5, 8, 9]. ONSD increments in cases of intracranial hypertension are mainly attributed to the fact that the intraorbital cerebrospinal fluid (CSF) space (i.e., the space between the dural sheath of the optic nerve and the nerve proper) is contiguous with the intracranial CSF space. In this study, the sonographic differentiation between the nerve proper and the CSF space was difficult. In order to standardize measurements and ensure their reproducibility, the “dark stripe” method was applied on all cases. The results suggested a statistically significant model-driven prediction equation of ICP derived by the ONSD measurements. The distribution of ONSD data exhibited a rather different pattern compared to PI data as the former also increased gradually but with a “step-like” manner which is in accordance with previous clinical studies [4, 5].

In this study, we have presented for the first time to the best of our knowledge experimental data regarding the sonographic consensual PLR test. The latter is an established neuromonitoring tool when performed by means of standard pupillometry [18]. The present results showed that the pupil constriction velocity becomes lower (the pupil's reaction to light becomes more sluggish) with increased ICP values and this occurs just before the occurrence of brain tamponade, CCA, or Cushing reaction, which is on accordance with previous reports (Figure 8) [18]. This sonographic method is not yet established in clinical neuromonitoring. The sonographic PLR test has many limitations as it is affected by various factors such as the depth of anesthesia and mechanical ventilation; however, the method has no real alternatives when visual access to the pupil is impossible, and its results are self-explanatory. The method is difficult in unstable gaze. In cases of severe facial trauma, extreme care should be taken to avoid additional damage to the globe and other tissues. The method can be combined with ONSD measurements to evaluate a potentially catastrophic intracranial process [19]. Interestingly, the distribution of pupil constriction velocity data (Figures 7(a) and 8(a)) resembled the pattern of the distribution of ONSD data (a “step-like” manner of distribution). This observation may strengthen the rationale behind a combined ONSD/PLR sonographic method and its relevance in neurocritical monitoring; however, further larger studies are clearly required to study the aforementioned suggestions. Finally, the combination of all studied ultrasound indices in one model-driven prediction equation of ICP was not possible due to the small size of the study group and the limited total number of ultrasound measurements. This remains a future research target of our group. Despite the many inherent and technical limitations of the presented experimental model, our results showed a clear relation of the studied ultrasound neuromonitoring indices with invasive ICP values in this experimental model of epidural hematoma

## 5. Conclusion

In conclusion, Doppler derived PI, ONSD measurements, and sonographically determined pupil constriction velocity (derived from a consensual PLR test) were all significantly related to invasive ICP increments in an experimental model of epidural hematoma. Statistically significant prediction models of ICP derived from all three ultrasound methods were registered. This observation may further support the rationale of pursuing the concept of a holistic ultrasound protocol (combination of various ultrasound neuromonitoring indices on the same monitoring platform) in the ICU [27]. Undeniably, further research work is clearly necessary to explore the role of ultrasound in modern neurocritical practice.

## Figures and Tables

**Table 1:** Ultrasound and other parameters in the study group (*n* = 20 rabbits).

Variable	Baseline		Volume of epidural hematoma = 1 mL	
	Mean (95% CI)	SD	Mean (95% CI)	SD
Intracranial pressure (mm Hg)	5.85 (5.57 to 6.12)	±0.58	42.20 (41.34 to 43.05)*	±1.82
Systolic blood pressure (mm Hg)	83.40 (80.78 to 86.01)	±31.20	199.30 (195.28 to 203.31)*	±8.57
Diastolic blood pressure (mm Hg)	49.10 (48.70 to 49.49)	±0.85	103.40 (101.31 to 105.48)*	±4.45
Pulsatility index (PI)	0.70 (0.64 to 0.76)	±0.13	Oscillating flow (PI > 7)*	
Optic nerve sheath diameter (mm)	1.73 (1.63 to 1.83)	±0.22	2.78 (2.64 to 2.92)*	±0.30
Maximum pupillary diameter (mm)	2.29 (2.24 to 2.33)	±0.10	2.33 (2.26 to 2.40)	±0.15
Minimum pupillary diameter (mm)	1.59 (1.54 to 1.63)	±0.10	2.33 (2.26 to 2.40)	±0.15
Pupil constriction velocity (cm/s)	0.089 (0.084 to 0.098)	±0.10	0.0 (fixed pupil)*	±0

*The differences between values of the neuromonitoring indices at baseline versus at volume of epidural hematoma = 1 mL were all statistically significant (paired *t*-test; *P* < 0.0001).

## References

[1] Raboel PH, Bartek J, Andresen M (2012). Intracranial pressure monitoring: invasive versus non-invasive methods-a review. *Critical Care Research and Practice*.

[2] Güiza F, Depreitere B, Piper I, Van den Berghe G, Meyfroidt G (2013). Novel methods to predict increased intracranial pressure during intensive care and long-term neurologic outcome after traumatic brain injury: development and validation in a multicenter dataset. *Critical Care Medicine*.

[3] Rosenberg JB, Shiloh AL, Savel RH, Eisen LA (2011). Non-invasive methods of estimating intracranial pressure. *Neurocritical Care*.

[4] Karakitsos D, Soldatos T, Gouliamos A (2006). Transorbital sonographic monitoring of optic nerve diameter in patients with severe brain injury. *Transplantation Proceedings*.

[5] Soldatos T, Karakitsos D, Chatzimichail K, Papathanasiou M, Gouliamos A, Karabinis A (2008). Optic nerve sonography in the diagnostic evaluation of adult brain injury. *Critical Care*.

[6] Kochanek PM, Carney N, Adelson PD (2012). Guidelines for the acute medical management of severe traumatic brain injury in infants, children, and adolescents-second edition. *Pediatric Critical Care Medicine*.

[7] Czosnyka M, Matta BF, Smielewski P, Kirkpatrick PJ, Pickard JD (1998). Cerebral perfusion pressure in head-injured patients: a noninvasive assessment using transcranial Doppler ultrasonography. *Journal of Neurosurgery*.

[8] Blaivas M, Theodoro D, Sierzenski PR (2003). Elevated intracranial pressure detected by bedside emergency ultrasonography of the optic nerve sheath. *Academic Emergency Medicine*.

[9] Hansen HC, Helmke K (1996). The subarachnoid space surrounding the optic nerves: an ultrasound study of the optic nerve sheath. *Surgical and Radiologic Anatomy*.

[10] Sargsyan AE, Hamilton DR, Melton SL (2009). Ultrasonic evaluation of pupillary light reflex. *Critical Ultrasound Journal*.

[11] Nelson RJ, Czosnyka M, Pickard JD (1992). Experimental aspects of cerebrospinal hemodynamics: the relationship between blood flow velocity waveform and cerebral autoregulation. *Neurosurgery*.

[12] Barzo P, Doczi T, Csete K, Buza Z, Bodosi M (1991). Measurements of regional cerebral blood flow and blood flow velocity in experimental intracranial hypertension: infusion via the cisterna magna in rabbits. *Neurosurgery*.

[13] De Bray JM, Saumet JL, Berson M, Lefteheriotis G, Pourcelot L (1992). Acute intracranial hypertension and basilar artery blood flow velocity recorded by transcranial Doppler sonography: an experimental study in rabbits. *Clinical Physiology*.

[14] Ungersbock K, Tenckhoff D, Heimann A (1995). Transcranial Doppler and cortical microcirculation at increased intracranial pressure and during the Cushing response: an experimental study on rabbits. *Neurosurgery*.

[15] Poularas J, Karakitsos D, Kouraklis G (2006). Comparison between transcranial color doppler ultrasonography and angiography in the confirmation of brain death. *Transplantation Proceedings*.

[16] Karakitsos D, Poularas J, Karabinis A, Dimitriou V, Cardozo A, Labropoulos N (2011). Considerations for the utilization of transcranial Doppler sonography in the study of progression towards cerebral circulatory arrest. *Intensive Care Medicine*.

[17] Douzinas EE, Kostopoulos V, Kypriades E (1999). Brain eigenfrequency shifting as a sensitive index of cerebral compliance in an experimental model of epidural hematoma in the rabbit: Preliminary Study. *Critical Care Medicine*.

[18] Taylor WR, Chen JW, Meltzer H (2003). Quantitative pupillometry, a new technology: normative data and preliminary observations in patients with acute head injury—Technical note. *Journal of Neurosurgery*.

[19] Sargsyan AE, Hamilton DR, Melton SL (2009). Ultrasonic evaluation of pupillary light reflex. *Critical Ultrasound Journal*.

[20] Scott M, Flaherty D, Currall J (2013). Statistics: are we related?. *Journal of Small Animal Practice*.

[21] Scott M, Flaherty D, Currall J (2013). Statistics: using regression models. *Journal of Small Animal Practice*.

[22] Chesnut RM, Temkin N, Carney N (2012). A trial of intracranial-pressure monitoring in traumatic brain injury. *The New England Journal of Medicine*.

[23] Lindegaard K-F, Grolimund P, Aaslid R, Nornes H (1986). Evaluation of cerebral AVM’s using transcranial Doppler ultrasound. *Journal of Neurosurgery*.

[24] Giller CA, Hodges K, Batjer HH (1990). Transcranial Doppler pulsatility in vasodilation and stenosis. *Journal of Neurosurgery*.

[25] Lindegaard KF, Newell DW, Aaslid R (1992). Indices of pulsatility. *Transcranial Doppler*.

[26] Czosnyka M, Richards HK, Whitehouse HE, Pickard JD (1996). Relationship between transcranial Doppler-determined pulsatility index and cerebrovascular resistance: An Experimental Study. *Journal of Neurosurgery*.

[27] Lumb PD, Karakitsos D (2014). Ocular ultrasound in the intensive care unit. *Critical Care Ultrasound*.

